# Role of P2X Purinoceptor 7 in Neurogenic Pulmonary Edema after Subarachnoid Hemorrhage in Rats

**DOI:** 10.1371/journal.pone.0089042

**Published:** 2014-02-12

**Authors:** Sheng Chen, Zhigang Zhu, Damon Klebe, Hetao Bian, Paul R. Krafft, Jiping Tang, Jianmin Zhang, John H. Zhang

**Affiliations:** 1 Department of Neurosurgery, Second Affiliated Hospital, School of Medicine, Zhejiang University, Zhejiang, China; 2 Department of Physiology and Pharmacology, Loma Linda University, Loma Linda, California, United States of America; University of South Florida, United States of America

## Abstract

**Introduction:**

Neurogenic pulmonary edema (NPE) is an acute and serious complication after subarachnoid hemorrhage (SAH) with high mortality. The present study aimed to test the therapeutic potential of brilliant blue G (BBG), a selective P2X purinoceptor 7 (P2X7R) antagonist, on NPE in a rat SAH model.

**Methods:**

SAH was induced by endovascular perforation. 86 Sprague-Dawley rats were randomly divided into sham, vehicle-, or BBG-treatment groups. Mortality, body weight, SAH grading, neurological deficits, NPE clinical symptoms, and pulmonary index were measured at 24 hours following SAH. Western blot, gelatin zymography, lung histopathology, and immunofluorescence staining were performed in the left lung lobe to explore the underlying mechanisms at 24 hours post-surgery.

**Results:**

The incidence of clinical symptoms was correlated with pulmonary index. P2X7R and the marker of alveolar type I epithelial cells (the mucin-type glycoprotein T1-α) immunoreactivities were generally co-localized. BBG administration decreased mature interleukin-1β, myeloperoxidase, and matrix metallopeptidase-9 activation, but increased tight junction proteins, such as ZO-1 and occludin, which ameliorated pulmonary edema via anti-inflammation and improved neurological deficits.

**Conclusion:**

P2X7R inhibition prevented NPE after SAH by attenuating inflammation. Thus, BBG is a potential therapeutic application for NPE after SAH and warrants further research.

## Introduction

Subarachnoid hemorrhage (SAH) is a major cause of permanent neurological damage [1]–[3]. Death and disability not only directly results from the initial hemorrhage and secondary neurological complications [4], but also from non-neurological medical complications. Non-neurological medical complications, including cardiac, pulmonary, gastrointestinal, renal, and hematological complications, cause an equal proportion of deaths as neurological complications [5].

Neurogenic pulmonary edema (NPE) is an increase in interstitial and alveolar fluid, which may occur within 3 days after SAH. The incidence of NPE following the central nervous system (CNS) injury ranges from 11% to 71%, based on post-mortem examinations [6], [7]. SAH patients that develop NPE have a higher mortality rate of approximately 10% [8]. Although NPE was recognized for over a century, it is still underappreciated in the clinical arena. Two different mechanisms of NPE likely coexist. One is a hemodynamic mechanism, due to an increase in pulmonary vascular pressure. The other is an ‘inflammatory’ mechanism, leading to increase in pulmonary capillary permeability. Reducing pulmonary inflammation after experimental SAH was effective in preventing acute SAH-mediated lung injury [9], although effective NPE management strategies have not been established. The P2X purinoceptor 7 (P2X7R) is an adenosine triphosphate (ATP)-gated ion channel, which is widely known for their proinflammatory reaction, in that it triggers inflammasome and induces the release of interleukins, including interleukin (IL)-1β, tumor necrosis factor-α and cycloxygenase-2. P2X7R is expressed on several lung cell types, e.g., type I alveolar epithelial cells [10], pulmonary endothelia [11], and resident cells of the immune system [12], [13]. The wide distribution of P2X7R in the lung makes it a particularly strong research interest in the pathophysiology of acute and chronic lung inflammation [13], [14]. P2X7R inhibition provides anti-inflammatory effect in several lung diseases, including smoke, pulmonary fibrosis and asthma [14]–[16]. However, the role of the P2X7R in SAH-induced NPE remains poorly understood. Brilliant Blue G (BBG) is a selective noncompetitive antagonist of the P2X7R, which has been described in SAH, ischemia, and traumatic brain injury with therapeutic effect [17]–[19]. Recently, BBG reduced IL-1β secretion in co-cultures of lung epithelialand macrophage cell lines [20]. BBG is a selective rat P2X7R antagonist that is well-tolerated by the human body [21]. The purpose of this study was to characterize the P2X7R's involvement in NPE in an SAH rat model. We hypothesize that the P2X7R inhibition by BBG will attenuate lung inflammation, prevent lung-blood barrier disruption, and thus be a potential therapy for NPE after SAH.

## Materials and Methods

### Ethics Statement

All experiments were approved by the Institutional Animal Care and Use Committee (IACUC) of Loma Linda University. This study was strictly carried out according to the recommendations in the Guide for the Care and Use of Laboratory Animals of the National Institutes of Health. Adult Sprague-Dawley rats (weight about 280–320 g, Harlan, CA) were housed in a 12 hour light/dark cycle at a consistent temperature and humidity with free access to food and water.

### SAH Surgery

All animals were assigned to the following three groups: Sham (n = 18), SAH+ vehicle group (n = 31), and SAH+BBG group (n = 29). Eight animals with SAH grading ≤7 were excluded because of mild brain injury. All evaluations were blindly performed at 24 hours following SAH surgery.

A SAH rat model was utilized with slight modification, as we described previously [22], [23]. Briefly, animals were anesthetized with 3% isoflurane in 70/30% medical air/oxygen. A sharpened monofilament nylon suture was introduced into the stump of left external carotid artery and advanced through the internal carotid artery into the bifurcation of the anterior cerebral artery and the middle cerebral artery, where resistance was encountered. The filament was advanced 3 mm further to perforate the artery, then immediately withdrawn to cause perfusion. In the sham operation, the filaments were advanced until resistance without arterial perforation. Following surgery, the incision was closed, and the rats were allowed to recover on an electric heating blanket. According to the previous study[24], 30 mg/kg BBG or 2 ml saline (vehicle) was intraperitoneally administrated at 30 minutes after SAH surgery. 25 µg/kg Buprenorphine was subcutaneously administered immediately after surgery to relieved severe pain.

### SAH Grading Score

High-resolution pictures of the brain base were taken after euthanization for assessing SAH severity. As previously described, the SAH grading was blindly assessed according to the high-resolution pictures. The animals received a total score ranging from 0 to 18 based on extravasation of blood into the subarachnoid space [25].

### Neurological Evaluation

The modified Garcia test was blindly evaluated by a score ranging from 3 to 22. The evaluation consisted of seven tests, including: Spontaneous activity (0–3), symmetry in the movement of all four limbs (0–3), forepaw outstretching (0–3), climbing (1–3), body proprioception (1–3), response to whisker stimulation (1–3), and beam balance (0–4). The beam balance score was according to walking distance on a narrow wooden beam for 1 minute. The mean score of three consecutive trials in a 3-minute interval was calculated. Higher scores indicate greater function.

### The NPE Criteria

**Figure 1:**
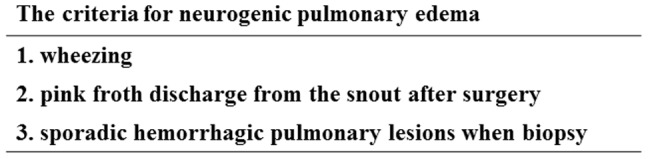
The criteria for neurogenic pulmonary edema.

The NPE was diagnosed by wheezing, a pink froth discharge from the snout, and sporadic pulmonary hemorrhagic lesions after biopsy (Fig. 1). The incidence of NPE was calculated in each group. Pulmonary edema was evaluated by the pulmonary index (P-index) according to a previous study [26].

P-index (%)  =  100× wet lung weight/body weight. Thus, the rats' weights were controlled between 280 g and 320 g before surgery.

### Western Blot Analysis

Western blot analysis was performed using the left lung [27]. Briefly, lung tissue was homogenized, and aliquots of each fraction were used to measure the protein concentration of each sample using a detergent compatible assay (Bio-Rad, Hercules, CA). Equal amounts of protein samples (40 µg) were subjected to electrophoresis in an SDS-PAGE gel and transferred onto a nitrocellulose membrane. Membranes were then blocked with blocking buffer for two hours, followed by incubation with the primary antibodies overnight at 4°C: rabbit polyclonal anti- IL-1β antibody (1∶1000) (Millipore Biosciences Research Reagents SBU, Temecula, CA), mouse monoclonal anti-myeloperoxidase (MPO, 1∶500), rabbit polyclonal anti-ZO-1 (1∶1000) and mouse monoclonal anti-Occludin (1∶1000) (Santa Cruz Biotechnology, Santa Cruz, CA). For loading control, β-Actin (1∶3000) (Santa Cruz Biotechnology, Santa Cruz, CA) was blotted on the same membranes. Immunoblots were processed with appropriate secondary antibodies (1∶2000) (Santa Cruz Biotechnology, Santa Cruz, CA) for one hour under room temperature. Blot bands were detected with a chemiluminescence reagent kit (ECL Plus; Amersham Bioscience, Arlington Heights, IL). The bands were quantified by densitometry with Image J software.

### Gelatin Zymography

Matrix metallopeptidase (MMP)-9 activity was measured by gelatin zymography according to the manufacturer's instruction. Equal amounts of protein (30 µg) were loaded on a 10% zymogram (gelatin) gels with zymography sample buffer (Invitrogen, Grand Island, NY). After electrophoresis, gels were washed with renature buffer (Bio-Rad, Hercules, CA) for one hour at room temperature and were incubated in developing buffer (Bio-Rad, Hercules, CA) at 37°C for three days for maximum sensitivity. Gels were stained with 0.5% coomassie blue G-250 for one hour and then were destained with the same buffer but without coomassie blue G-250. Human MMP-9 (Millipore, Billerica, MA) was used as the gelatinase standard. The densities of bands were measured using the image J software.

### Lung Histopathology and Immunofluorescence Staining

Under deep anesthesia, rats were intracardially perfused with 300 ml of ice-cold phosphate buffered saline (PBS, pH 7.4), followed by 100 ml of 10% buffered formalin. Lungs were harvested and then fixed for 24 hours and were cryopreserved in 30% sucrose and frozen in tissue-freezing media (Triangle Biomedical Sciences). Ten-micrometer coronal lung sections were cut in a cryostat (Leica CM3050S, Buffalo Grove, IL).

Lung specimens on glass microscope slides were stained with hematoxylin and eosin (H&E) and examined using light microscopy [28]. Immunofluorescence staining was performed as previously described with minor modifications [29], using the marker for alveolar type I epithelial cells (T1-α) (1∶50) (Acris Antibodies, San Diego, CA) and P2X7R (1∶50) (Santa Cruz Biotechnology, Santa Cruz, CA). Lung sections were incubated with a mixture of the above mentioned primary antibodies over night at 4°C, followed by a mixture of FITC- and Texas red-conjugated secondary antibodies (Jackson Immunoresearch, West Grove, PA) for 2 hours at room temperature. Slides were mounted with VECTASHIELD mounting medium with DAPI (Vector laboratory, Burlingame, CA). Microphotographs were snapped with a fluorescent microscope (Olympus OX51).

### Statistical Analysis

Data was expressed as mean ± standard error of the mean (SAH grading, P-index, Western blot, and Gelatin zymography) or median±25th–75th percentiles (Neurological score). Analysis was performed using GraphPad Prism or SPSS 16.0 software. Statistical differences between two groups were analyzed using Student's unpaired, two-tailed t-test. Multiple comparisons were statistically analyzed with one-way analysis of variance (ANOVA) followed by Student-Newman-Keuls test. For neurological score, we used the Kruskal-Wallis One-way ANOVA on Ranks, followed by the Steel-Dwass multiple comparisons tests. *P*<0.05 was defined as statistically significance. Spearman's correlation coefficients were performed to assess the correlation between mature IL-1β and neurological score.

## Results

### Mortality, SAH Grade and body weight

**Figure 2:**
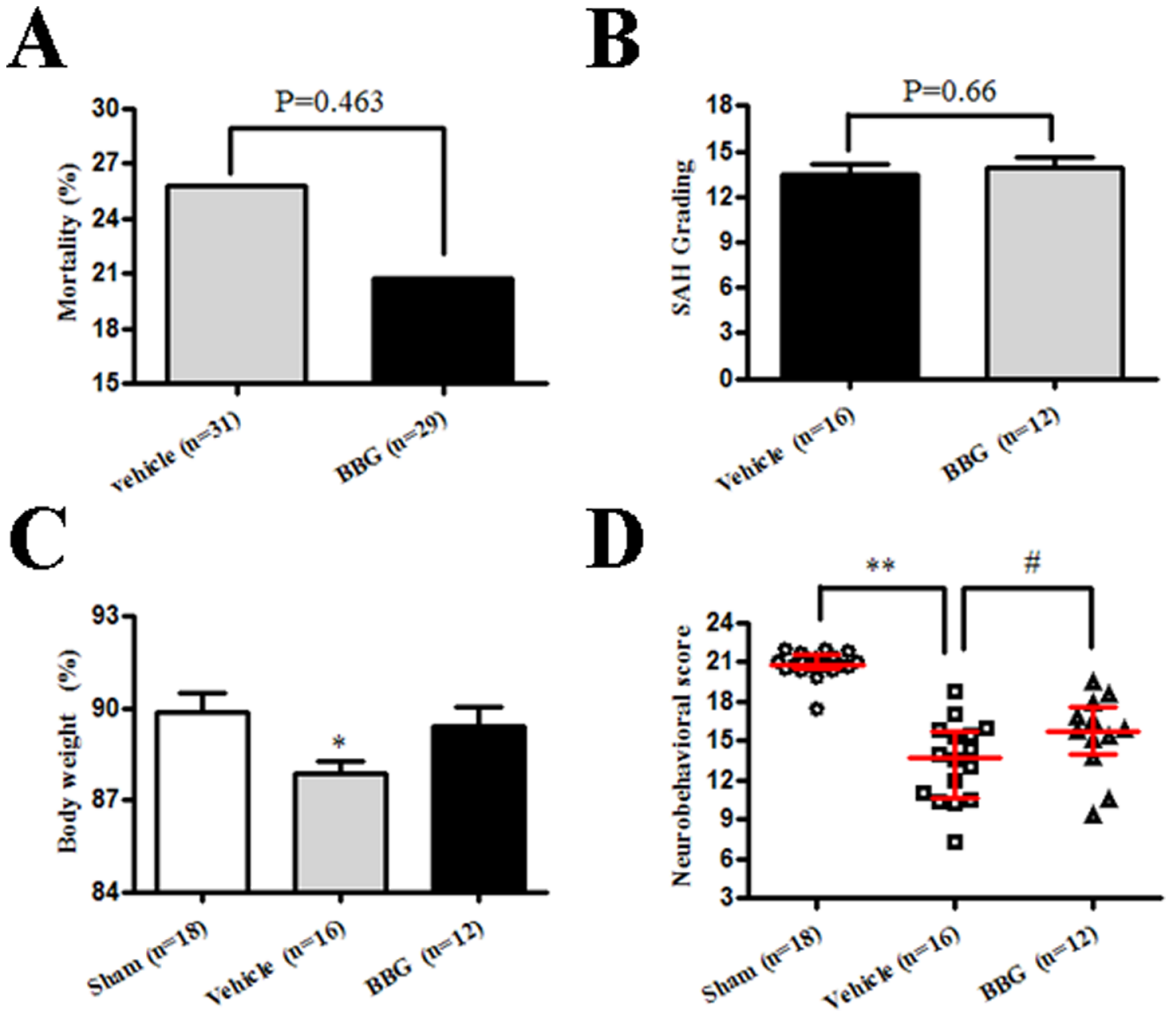
Mortality, SAH grading, body weight, and neurological score. A and B: Mortality and SAH grading, no significant difference between the SAH+vehicle group and the SAH+BBG group. C: SAH induced body weight loss, BBG administration increased body weight without a significant difference. D: BBG administration improved neurological deficits at 24 hours after SAH. B and C, Error bars represent mean ± standard error of the mean. D, Error bars represent median ±25th–75th interquartile percentiles. * *p*<0.05 vs. Sham; ** *p*<0.01 vs. Sham; # *p*<0.05 vs. SAH+vehicle group.

Fig. 2A showed no significant differences in mortality rate were observed between the SAH+vehicle group (25.81% [8 of 31 rats]) and SAH+BBG group (20.69% [6 of 29 rats]; *p* = 0.463 *vs.* SAH+vehicle group). Observed SAH grading score were similar between the SAH+vehicle group and SAH+BBG group (Fig. 2B). BBG administration failed to prevent body weight loss (*p*>0.05 *vs.* SAH+vehicle group) (Fig. 2C).

### Neurological score

Neurological tests revealed significant neurological deficits in the SAH compared to sham operated animals (*p*<0.05 *vs.* sham). Post-SAH administration of BBG significantly improved neurobehavioral deficits compared to vehicle animals at 24 hours after SAH (*p*<0.05 *vs.* SAH+vehicle group) (Fig. 2D).

### The incidence of NPE and the P-index

**Figure 3:**
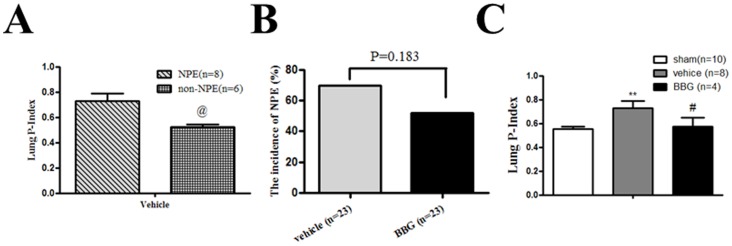
Change in the P-index at 24 hours after SAH. A: The P-index of NPE was significantly higher than non-NPE in SAH rats. @ *p*<0.05 vs. NPE; B: BBG decreased the incidence of NPE without reaching significance; C: BBG significantly reduced the P-index at 24 hours after SAH. Error bars represent mean ± standard error of the mean. * *p*<0.05 vs. Sham; ** *p*<0.01 vs. Sham; # *p*<0.05 vs. SAH+vehicle group.

In SAH+vehicle group, the animals were diagnosed with NPE if they presented wheezing and a pink foam discharge from their snout after surgery as well as sporadic hemorrhagic pulmonary lesions after biopsy. The P-index in NPE animals was 0.73 (*p*<0.01 *vs.* sham group), but only 0.53 in non-NPE group (Fig. 3A). The p-index of NPE was significantly higher than non-NPE in SAH rats (*p*<0.05). The incidence of NPE in SAH+vehicle group was 69.57%. BBG treatment decreased the incidence of NPE to 52.17% compared to vehicle administration (*p* = 0.183 *vs.* SAH+vehicle group) (Fig. 3B). In NPE rats after SAH, the average P-index in the SAH+BBG group was 0.57, which is lower than SAH+vehicle group (*p*<0.05) (Fig. 3C).

### BBG reduced inflammation, but prevented tight junction protein degradation in the left lung at 24 hours following SAH

**Figure 4:**
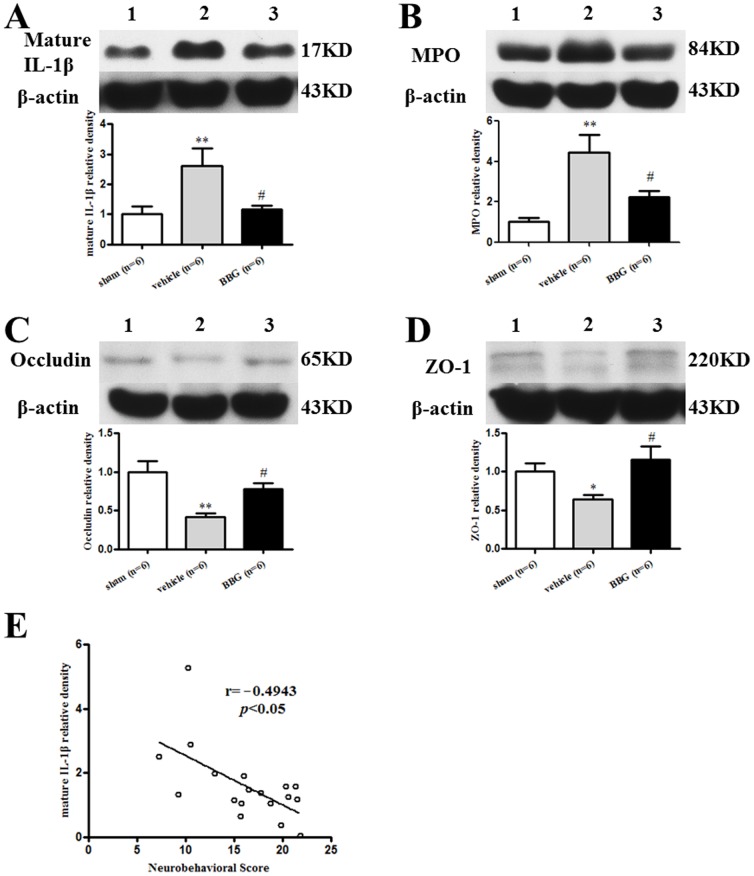
Quantitative analysis of mature IL-1β(A), MPO (B), Occludin (C), and ZO-1 (D) expressions 24 hours post-SAH. 1, sham group; 2, SAH+Vehicle group; 3, SAH+BBG group. n = 6 rats per group. Error bars represent mean ± standard error of the mean. * *p*<0.05 vs. Sham; ** *p*<0.01 vs. Sham; # *p*<0.05 vs. SAH+vehicle group. Correlation between mature IL-1β and neurobehavioral score (E).

**Figure 5:**
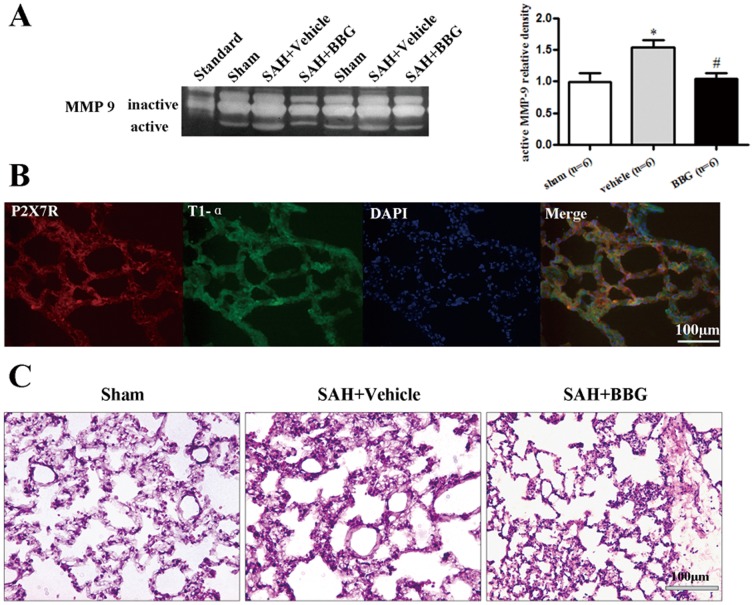
Lung histopathology at 24 hours after SAH. (A) Zymography assay for MMP-9 activity in the left lung in sham, vehicle, and BBG treated groups. (B) Representative microphotographs of immunofluorescence staining showing co-localization of T1-α (FITC/green), P2X7R (Texas red/red) and DAPI (blue). Scale bar = 100 µm. (C) H&E staining showed BBG administration ameliorated diffuse neutrophilic infiltration and swelling of the alveolar interstitium.

The mature IL-1β and MPO expression of left lung tissue in SAH+vehicle group increased by 259.8% and 443.9% when compared with the sham group (*p*<0.05), but only 116.8% and 222.7% elevation was detected in SAH+BBG group, respectively (*p*<0.05 *vs.* SAH+vehicle group, Fig. 4A and Fig. 4B). The expression of occludin and ZO-1 of lung tissue significantly decreased to 41.6% and 63.6% in SAH+vehicle group (*p*<0.05 *vs.* sham), while BBG administration reversed their expression compared to vehicle administration (*p*<0.05 *vs.* SAH+vehicle group; Fig.4C and Fig. 4D). The level of mature IL-1β is significantly correlation with neurobehavioral score (r = −0.4943, *p*<0.05, Fig. 4E). Active MMP-9 levels significantly increased after SAH when compared with the sham group, whereas BBG treatment decreased active MMP-9 levels compared to vehicle animals (*p*<0.05 *vs.* SAH+vehicle group; Fig. 5A).

### BBG ameliorated pulmonary edema in histology at 24 hours after SAH

P2X7R and T1-α immunoreactivities were generally co-localized, suggesting P2X7R is expressed in alveolar type I epithelial cells (Fig.5B). H&E staining depicted diffuse neutrophilic infiltration into the lung tissue and edema formation in the alveolar interstitium of the vehicle group, whereas BBG administration ameliorated pulmonary edema (Fig. 5C).

## Discussion

In this study, for first time, we diagnosed NPE in an animal study according to related clinical presentations, and the incidences of those symptoms are correlated with the P-index after SAH. Thus, this method may be a simple but effective means to characterize NPE in small animal studies. Furthermore, BBG, a selective P2X7R antagonist, inhibited pulmonary edema and eventually improved neurological deficits via attenuating inflammation and degradation of tight junction molecules.

Clinically, NPE is a difficult and usually exclusive diagnosis due to common presentations. In animal studies, P-index is proven to be a very sensitive indicator of the degree of pulmonary edema [26]. In the present study, NPE was diagnosed according to related clinical symptoms. For those rats who meet the above NPE criteria, P-index is higher than non-NPE rats in the vehicle group. The incidence detected by this method is close to data from prior post-mortem examinations [6]. Thus, this simple method may potentially be used for NPE diagnosis in experimental NPE studies.

The therapeutic strategy for NPE is based on the treatment for pulmonary edema [30]. The inflammatory response plays an important role in aggravating pulmonary edema associated with releasing MMPs that degrade tight junctions and impair the alveolar-capillary barrier [31]. Previously, Cobelens et al. reported SAH induced the influx of neutrophils and increased IL-1β in the lung [9]. We found active IL-1β increased in NPE animals after SAH [32]. Similar result was observed in the present study, with elevated IL-1β level 24 hours after SAH. Additionally, IL-1β induces MMP-9 secretion and activation via NF-κB signaling pathways, which conferred a barrier-damage effect [33], [34]. In turn, active MMP-9 could further drive IL-1β activation [35]. Moreover, MMP-9 alters the localization of tight junction components, such as claudin-1, occludin, and ZO-1, adversely affecting barrier function and eventually directly damaging respiratory epithelium in asthma [36], [37]. In our study, active MMP-9 was found significantly increased, whereas occludin and ZO-1 were decreased in left lung after SAH. Taken together, these findings supported that IL-1β is a key regulator of MMP-9 and induces blood-lung barrier disruption after SAH. Therefore, IL-1β can be a therapeutic target for NPE treatment.

Extracellular ATP is a danger-associated molecular marker, which rapidly increased after epithelial cell damage or lysis following mechanical stress, trauma, extracellular hypotonicity, oxidative stress, and infection [11]. It plays a critical role in the inflammation of the airways by acting on P2X7R [38]. The P2X7R is an ATP-gated cation channel in that sustained activation by extracellular ATP leads to rapid opening of a reversible plasma membrane pore permeable to molecular mass up to 900 Da [39]; and to release of the pro-inflammatory cytokine, IL-1β [19]. In a bleomycin-induced lung fibrosis murine model, ATP released from damaged lung cells leads to IL-1β maturation and lung fibrosis via P2X7R [16]. By LPS-challenge, lung influx of IL-1β and MMP-2 release was lower in P2X7R knockout mice compared to wild-type mice [13]. Additionally, P2X7R mediated NLRP3 inflammasome activation, which controls the production of mature IL-1β [40]. A selective P2X7R antagonist or P2X7R genetically silenced mice attenuated IL-1β release and airway neutrophil recruitment after exposure to tobacco smoke [15]. P2X7R may be involved in LPS-induced p65-Ser311 NF-κB phosphorylation [41], which suggests P2X7R antagonism may directly reduce MMP-9 via NF-κB pathway. In the present study, P2X7R is expressed on alveolar type I epithelial cells, which play vital roles in alveolar barrier damage and fluid homeostasis. BBG suppressed IL-1β release and neutrophil recruitment post-SAH in the lung, subsequently inhibited MMP-9 activation, and prevented the degeneration of tight junction proteins. Eventually, BBG decreased pulmonary edema and improved neurological deficits after SAH. Taken together, these lines of evidence indicate P2X7R may be a new therapeutic option for NPE treatment.

There are some limitations in the present study. First, our previous study encourages the application of BBG as a potential neuroprotective therapy for SAH. Therefore, the improved neurobehavioral score in this present study may because of the combination of directly neuroprotective effect or indirect by reducing NPE. Secondly, future studies are warranted to observe the new criteria as elements of a score (e.g. 1–3 depending on how many of the criteria are present) and perform a ROC analysis with the P-index. At last, we did not measure the change of P2X7R activity in the lung by using BBG. We expected that BBG would reduce an inward current by electrophysiology monitoring and decreased Ca^2+^ influx by imaging.

### Conclusion

We presented the first experimental study describing clinical symptoms for useful NPE diagnosis after SAH. Furthermore, P2X7R antagonism ameliorates NPE via its anti-inflammatory effects after SAH. BBG is a potential therapeutic application for NPE after SAH and warrants further research.
